# Hippocampal protein expression is differentially affected by chronic paroxetine treatment in adolescent and adult rats: a possible mechanism of “paradoxical” antidepressant responses in young persons

**DOI:** 10.3389/fphar.2013.00086

**Published:** 2013-07-08

**Authors:** Emily A. Karanges, Mohammed A. Kashem, Ranjana Sarker, Eakhlas U. Ahmed, Selina Ahmed, Petra S. Van Nieuwenhuijzen, Andrew H. Kemp, Iain S. McGregor

**Affiliations:** School of Psychology, University of SydneySydney, NSW, Australia

**Keywords:** antidepressant, paroxetine, proteomics, adolescent, hippocampus, rat

## Abstract

Selective serotonin reuptake inhibitors (SSRIs) are commonly recognized as the pharmacological treatment of choice for patients with depressive disorders, yet their use in adolescent populations has come under scrutiny following reports of minimal efficacy and an increased risk of suicidal ideation and behavior in this age group. The biological mechanisms underlying these effects are largely unknown. Accordingly, the current study examined changes in hippocampal protein expression following chronic administration of paroxetine in drinking water (target dose = 10 mg/kg for 22 days) to adult and adolescent rats. Results indicated age-specific changes in protein expression, with paroxetine significantly altering expression of 8 proteins in adolescents only and 10 proteins solely in adults. A further 12 proteins were significantly altered in both adolescents and adults. In adults, protein changes were generally suggestive of a neurotrophic and neuroprotective effect of paroxetine, with significant downregulation of apoptotic proteins Galectin 7 and Cathepsin B, and upregulation of the neurotrophic factor Neurogenin 1 and the antioxidant proteins Aldose reductase and Carbonyl reductase 3. Phosphodiesterase 10A, a signaling protein associated with major depressive disorder, was also downregulated (-6.5-fold) in adult rats. Adolescent rats failed to show the neurotrophic and neuroprotective effects observed in adults, instead displaying upregulation of the proapoptotic protein BH3-interacting domain death agonist (4.3-fold). Adolescent protein expression profiles also suggested impaired phosphoinositide signaling (Protein kinase C: -3.1-fold) and altered neurotransmitter transport and release (Syntaxin 7: 5.7-fold; Dynamin 1: -6.9-fold). The results of the present study provide clues as to possible mechanisms underlying the atypical response of human adolescents to paroxetine treatment.

## Introduction

Selective serotonin reuptake inhibitors (SSRIs) are commonly recognized as the pharmacological treatment of choice for patients with depressive disorders, and have dominated the antidepressant market since the late 1990s (Mant et al., 2004). SSRIs are generally preferred over the older tricyclic antidepressants (TCAs) and monoamine oxidase inhibitors (MAOIs) due to their more favorable side-effect profile, ease of use, relative safety in overdose, and comparable efficacy (Fava and Kendler, 2000). However, the safety and efficacy of SSRIs for the treatment of adolescent depression is a topic of considerable debate. Various studies have suggested that SSRIs, with the possible exception of fluoxetine (Whittington et al., 2004), have minimal efficacy in young people (Cheung et al., 2005; Tsapakis et al., 2008) and are associated with increased risk of psychiatric adverse effects including suicidal ideation and behavior (Hammad et al., 2006; Stone et al., 2009). Nevertheless, the association between SSRIs and suicidality remains uncertain, with population studies suggesting a negative relationship between SSRI use and youth suicide (Gibbons et al., 2007). In addition, the neural mechanisms underlying these age-specific effects are unknown.

SSRIs act by selectively binding to and inhibiting the serotonin transporter (SERT), which is responsible for the reuptake of synaptic serotonin (5-hydroxytryptamine; 5-HT) (Frazer, 1997). While this inhibition of reuptake leads to an acute increase in synaptic 5-HT (Bosker et al., 1995) SSRIs generally take 2–4 weeks to reach full efficacy (Delago, 2004), thus other mechanisms appear to be involved in their antidepressant actions. Various neuroadaptations correlate with the onset of antidepressant efficacy, including desensitization and/or downregulation of 5-HT_1A_, 5-HT_1B_ and 5-HT_2C_ receptors (Artigas et al., 2001; Yamauchi et al., 2004); upregulation of dopaminergic tone (Di Giovanni et al., 2008); increases in neurogenesis (Feldmann et al., 2007); and stimulation of key transcription factors such as cAMP-response element binding protein (CREB) and trophic factors such as brain-derived neurotrophic factor (BDNF) (Hyman, 2006).

However, it is not clear whether these neural effects also occur in the developing brain. Adolescence is a critical developmental period characterized by high levels of neuroplasticity and neural reorganization. These processes could conceivably be disrupted by antidepressant medications, producing atypical behavioral and emotional effects. This hypothesis was explored in the current study through the use of an adolescent animal model. The adolescent period in male rats spans from approximately post-natal day (PND) 28 until PND 55 (Spear, 2007). During this period rats display adolescent-typical behavioral and neural characteristics including elevated risk-taking and social interaction (Crews et al., 2007); increased myelination, synaptic pruning and neurogenesis (Paus, 2005; Hodes et al., 2009); general upregulation of serotonergic tone (Murrin et al., 2007); and heightened physiological responses to stress (McCormick and Mathews, 2010). Interestingly, adolescent rodents show a behavioral response to SSRIs that tends to reflect the atypical reaction in adolescent clinical populations. Peri-pubertal mice treated with fluoxetine display a paradoxical anxiogenic response (Oh et al., 2009) and fail to increase struggle on the forced swim test (FST) (Mason et al., 2009), suggesting limited efficacy in juvenile populations. We have previously reported similar behavioral findings in adolescent rats treated with paroxetine (PRX), and further demonstrated that these atypical behavioral responses are accompanied by opposite serotonergic and dopaminergic neuroadaptations to those observed in adult rats (Karanges et al., 2011).

Located in the medial temporal lobe, the hippocampus connects to the amygdala and prefrontal cortex, regions involved in control of emotion and cognition respectively, and also plays a role in regulation of hypothalamic-pituitary-adrenal (HPA) axis function (Duman and Monteggia, 2006). Furthermore, the hippocampus appears particularly susceptible to stress-induced morphological changes (McEwen, 2001). Hippocampal atrophy has been observed in patients with major depressive disorder (MDD) and animal models of chronic stress, with these effects often reversed by antidepressant treatment (McKernan et al., 2009). Thus, the hippocampus represents a key site for depressogenic neural changes and corresponding restorative antidepressant action.

In order to further explore developmental differences in the antidepressant response, and to reveal potential mechanisms underlying the atypical response in adolescents, the present study investigated differential changes in hippocampal protein expression following chronic PRX administration to adolescent and adult rats.

## Materials and methods

### Subjects

The subjects were 32 adolescent (PD 28; 77–118 g) and 32 adult (PD 70; 313–447 g) experimentally naïve male albino Wistar rats (Animal Resource Centre, Perth, Australia), housed in age- and treatment-matched groups (adults: 4/cage; adolescents: 8/cage) with food and water freely available at all times. Home cages were kept in a temperature- (22 ± 2°C) and humidity-controlled colony room with 12-h reverse light cycle (lights on at 21:00 h). Care was taken to minimize the number of animals used and their suffering. All experimentation was approved by the University of Sydney Animal Ethics Committee in accordance with the *Australian Code of Practice for the Care and Use of Animals for Scientific Purposes*.

### Drug treatment and experimental design

Rats were randomly divided into four groups (*n* = 16): Adult/PRX, Adult/Control, Adolescent/PRX and Adolescent/Control, such that half of each developmental cohort received PRX solution in place of standard drinking water. Administration via drinking water has previously been successfully employed in our laboratory (Thompson et al., 2004) and was chosen due to the short half-life of PRX (Owens et al., 2000). Paroxetine hydrochloride was obtained from Sequoia Research Biochemicals (Pangbourne, UK) and administered at a target dose of 10 mg/kg. The dose was selected on the basis of previous literature showing antidepressant and neurochemical effects of this dose in adult rats (Carlson et al., 1996; Sillaber et al., 2008), and to approximate therapeutic plasma concentrations in humans (DeVane, 1999). Prior to target dose administration, a 5 mg/kg half-dose was administered for 3 days in accordance with recommendations for the treatment of children and adolescents with SSRIs (Fleming, 2007). Taking body weight and fluid intake into account, PRX was dissolved in water at the appropriate concentration, adjusted continuously, and administered for 22 days in light-proof bottles in place of standard drinking water. The concentrations of PRX present in plasma at the end of this 22 day period have been previously reported (Karanges et al., 2011). Although both adolescents and adults exhibit concentrations well within the therapeutic range (10–600 ng/ml; DeVane, 1999), they were significantly lower in adolescents (105.19 ± 17.49 nmol/l) than in adults (308.59 ± 80.52 nmol/l) (*p* < 0.05).

### Euthanasia and sample collection

On day 22 of drug administration, rats were euthanized by decapitation. Brains were rapidly removed and the hippocampus manually dissected out over ice and snap-frozen in liquid nitrogen. Samples were stored at −80°C until required for proteomic analysis.

### Two-dimensional gel electrophoresis (2DE) proteomic analysis

The hippocampi of 24 rats (*n* = 6/group) were used for proteomic analysis. Protein extraction and analysis was performed as previously described (Ahmed et al., 2012; Kashem et al., 2012), using a protocol optimized for cytosolic proteins. Hippocampal tissue was homogenized in buffer consisting of 7 M urea, 2 M thiourea, 1% C7bZO and 40 mM Tris, sonicated and pelleted. The supernatant was reduced and alkylated in 5 mM tributylphosphine (TBP) and 10 mM acrylamide monomer and quenched using 10 mM dithiothreitol (DTT). The mixture was acidified (pH approximately 6.0) with citric acid and precipitated by acetone. The precipitant was pelleted, air-dried and resuspended in 7 M urea, 2 M thiourea and 1% C7bZO.

Protein concentration was determined by the Bradford method (Bradford, 1976). Immobilized pH gradient strips (IPG strips; 11 cm, pH 4–7) were rehydrated with samples containing 400 μg protein for 6 h at room temperature. Re-hydrated strips were focused using the ElectrophyoretIQ^3^ system (Proteome Systems Ltd, Australia) for a total of 120 kVh. IPG strips were equilibrated using ProteomIQ™ SDS equilibration buffer and loaded onto SDS-PAGE gels (8–16%, 10 × 15 cm) for second dimension separation by the ElectrophoretIQ^3^ system (30 mA/gel, 25°C for 110 min; Proteome Systems, Australia). Gels were fixed with methanol [25% (v/v)] and acetic acid [10% (v/v)] and stained using colloidal Coomassie Blue for spot visualization.

A total of 48 gels were prepared (*n* = 6/group, in duplicate). Gels were digitized at 400 dots per inch using a transmissive flatbed scanner (UMAX) and analysed using Phoretix 2D Expression software (Non-linear Dynamics Ltd., UK). To assist with comparison and reduce within group variation, averaged gels were created using the 12 gels for each group, with averaging parameters set at 70% (i.e., for a spot to appear in the averaged gel, it must occur in 70% of all gels in the group). Normalized spot volumes were calculated as the natural log of spot pixel value, allowing for calibration of data between different sample runs and correction for experimental variation. Fold changes in protein expression were calculated as the ratio of the normalized spot volume for the treatment group and age-matched control. Independent-samples *t*-tests (*p* < 0.05) were performed using the normalized volume of spots from age-matched Control and Paroxetine-treated groups.

Protein spots identified as significantly altered were destained in 50 mM ammonium bicarbonate and acetonitrile (ACN) solution (60:40) for 1 h at room temperature. Spots were dried and rehydrated in a tryptic digest solution (12 ng/μl porcine sequencing grade trypsin [Promega, USA] in 50 mM NH_4_HCO_3_) at 4°C for 1 h. Residual digest solution was removed and the gel pieces suspended in 50 mM NH_4_HCO_3_ for 3 h at 37°C. The peptide mixtures were purified using C_18_ Perfect Pure Tips (Eppendorf, Germany), and the sample eluted onto a MALDI sample plate with 2 μl matrix solution [5 mg/ml solution of α-cyano-4-hydroxy-cinnamic acid (Sigma-Aldrich, USA) in 70% ACN/0.1% v/v trifluoroacetic acid (TEA)] and allowed to air dry. Samples were analysed by matrix assisted laser desorption/ionisation time-of-flight mass spectrometry (MALDI-TOF MS) using an Applied Biosystems Voyager with MALDI source (Sydney University Proteome Research Unit, SUPRU). The mass fingerprint data (MALDI spectra) were searched against the Swiss-Prot database using the MASCOT search engine (http://www.matrixscience.com). The probability-based MOWSE (molecular weight search) algorithm was used to determine protein identities from peptide fragment masses (Pappin et al., 1993). Positive protein identifications were based on a significant MOWSE score (>54% peptide match, *Rattus* database), in conjunction with matched pI and molecular weight values as estimated from 2D gels.

## Results

**Figure 1:**
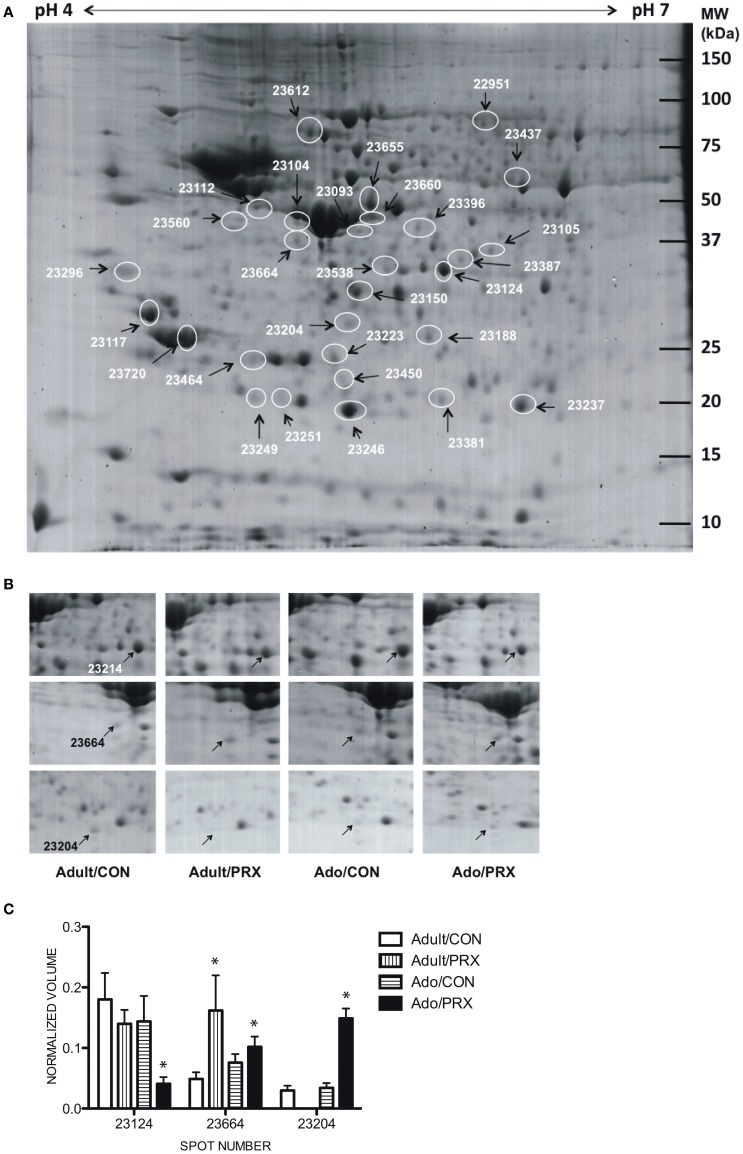
**Results of proteomics conducted on the hippocampus of adult and adolescent rats chronically treated with paroxetine (10 mg/kg). (A)** A representative 2-DE gel image of a hippocampal protein sample from an Adolescent/Control rat. Approximately 400 μg of protein was loaded onto an IPG strip (pH 4–7) followed by 2-dimensional electrophoresis. Experimental masses are located on the Y-axis and pI's on the X-axis. Spot numbers are given in Table 1. **(B)** Cropped 2-DE gel images of 3 hippocampal protein spots from each of the 4 experimental groups: Adult/PRX, Adult/Control, Adolescent/PRX and Adolescent/Control. PRX downregulated Protein kinase C (PKC; spot 23124; top panel) and upregulated BH3-interacting domain death agonist (BID; spot 23204; bottom panel) in adolescents only. Tryptophan-5-hydroxylase 1 (TPH1; spot 23664; middle panel) was upregulated in 4.3-fold adult rats, but only 1.6-fold in adolescents. **(C)** Normalized volumes of Protein kinase C, Tryptophan-5-hydroxylase and BH3-interacting domain death agonist. ^*^p < 0.05.

The averaged gels for Adult/PRX, Adult/Control, Adolescent/PRX and Adolescent/Control displayed 550, 563, 557, and 540 spots respectively. Thirty-one protein spots were differentially regulated in adolescent and 45 in adult PRX-treated rats (*p* < 0.05), with 14 of these spots significantly altered by PRX in both adults and adolescents. In adolescents, 11 spots were increased more than 2-fold, and 10 were decreased more than 2-fold. In adults, these values were 8 and 21 respectively. These differentially expressed spots were identified as 30 different proteins using MALDI-TOF MS, with 8 proteins significantly altered in adolescents only, 10 proteins in adults only, and 12 proteins altered in both age groups. A list of proteins differentially regulated by PRX in adult and adolescent rats, and those changed in both cohorts, is shown in Table 1. Figure 1A shows a representative 2-DE gel image of protein expression in the hippocampus of an Adult/Control rat. Magnified images of protein spots 23124 (Protein kinase C), 23664 (Tryptophan-5-hydroxylase 1) and 23204 (BH3-interacting domain death agonist) and normalized spot volumes from each of the 4 groups are also shown (Figures 1B,C respectively).

**Table 1:** **Differentially expressed proteins in the hippocampus of adult and adolescent paroxetine- (PRX) treated rats (*n* = 6/group)**.

**Spot number**	**Protein name**	**Accession number**	**pI**	**MW**	**Matched**	**Sequence Cover (%)**	**Fold change**	
							**Adult**	**Adolescent**
**SIGNALLING PROTEINS**								
**Changed in adults and adolescents**								
23655	Phosphodiesterase 4A (PDE4A)	P54748	5.37	41	5	21	2.57^*^	2.80^*^
23112	Protein kinase A (PKA)	Q5BJR2	5.10	24	5	17	2.23^*^	2.20^*^
23387	Protein phosphatase 6, catalytic subunit (PP6c)	Q64620	5.64	35	4	16	-0.70^*^	-2.26^*^
23091	Guanine nucleotide-binding protein Gi, α 1 subunit (Gnai1)	P10824	5.66	35	6	31	2.11^*^	2.38^*^
23717	14-3-3 protein epsilon (14-3-3-ε)	P62259	4.63	29	5	22	1.51^*^	1.53^*^
**Changed in adults only**								
22951	Phosphodiesterase 10A (PDE10A)	Q9QYJ6	6.65	74	12	24	-6.48^*^	-1.44
23450	Rab28	Q6IN03	5.30	24	4	25	-1.80^*^	1.00
**Changed in adolescents only**								
23124	Protein kinase C (PKC)	Q568X9	6.01	33	10	19	-1.03	-3.12^*^
**OXIDATIVE STRESS PROTEINS**								
**Changed in adults only**								
23660	Carbonyl reductase 3 (CBR3)	Q9JJN7	5.59	30	7	26	3.51^*^	1.33
23093	Aldose reductase (AR)	P07943	6.28	35	5	31	3.09^*^	1.44
**APOPTOTIC PROTEINS**								
**Changed in adults only**								
23381	Galectin-7 (GAL7)	P97590	6.43	15	5	29	-3.81^*^	ND
23246	Cathepsin B (CTSB)	P00787	5.14	27	4	18	-1.58^*^	-1.28
**Changed in adolescents only**								
23204	BH3-interacting domain death agonist (BID)	Q9JLT6	4.82	22	4	37	ND	4.34^*^
**METABOLIC PROTEINS**								
**Changed in adults and adolescents**								
23538	Cytochrome P450 1B1 (CYP1B1)	Q9ESW3	5.58	25	6	34	-4.50^*^	-4.08^*^
23560	Tyrosine aminotransferase (TAT)	P04694	5.13	50	6	14	2.17^*^	2.22^*^
23251	Ferritin light chain 1 (FTL1)	P02793	5.88	20	5	33	2.23^*^	2.20^*^
23105	Guanosine monophosphate reductase 2 (GMPR2)	Q5FVP6	6.51	31	5	24	-2.32^*^	-2.19^*^
23612	5-Hydroxytryptamine receptor 3B (5-HT-3B)	Q9JJ16	5.34	50	6	24	1.89^*^	1.55^*^
23664	Tryptophan-5-hydroxylase 1 (TPH1)	P09810	6.30	51	6	15	4.32^*^	1.55^*^
**Changed in adults only**								
23188	Neurogenin-1 (NG1)	P70595	6.43	26	3	19	4.19^*^	1.40
23296	Phenylethanolamine-N-methyltransferase (PNMT)	P10937	4.94	21	4	21	-4.10^*^	1.40
23396	Choline-phosphate cytidylyltransferase A (CCTa)	P19836	6.58	41	8	25	-2.10^*^	1.40
23150	Quinone reductase 2 (QR2)	Q6AY80	6.90	26	6	27	-1.85^*^	-1.17
**Changed in adolescents only**								
23464	Interferon γ inducible protein 30	Q499T2	4.77	27	5	31	ND	3.85^*^
23249	Phospholipase C (δ-1) (PLCδ1)	P10688	5.36	27	7	31	-1.13	-1.63^*^
**CYTOSKELETAL PROTEINS**								
**Changed in adults and adolescents**								
23237	Dynamin-1 (DYN1)	P21575	6.97	22	4	22	-2.63^*^	-6.89^*^
**Changed in adolescents only**								
23223	Tropomyosin-4 (TM4)	P09495	4.66	28	4	18	-1.23	-2.25^*^
23437	Pannexin-1 (PX1)	P60570	6.34	40	6	20	-1.00	-1.79^*^
**CALCIUM-REGULATING PROTEINS**								
**Changed in adolescents only**								
23104	Syntaxin 7 (STX7)	O70257	6.02	29	5	23	ND	5.74^*^
23720	Calcium binding protein 1 (CBP1)	O88751	4.77	25	6	31	1.42	1.53^*^

*Significant alterations in protein expression in the hippocampus of adolescent and adult PRX-treated rats were established by comparison with age-matched VEH-treated averaged gels. Protein spots of interest were excised and subjected to MALDI-TOF MS. Positive protein identifications were based on a significant MOWSE score (>54%), with matched pI and M_w_ values*.

*pI, isoelectric point; MW, molecular weight; ND, not detectable*.

* *p < 0.05, Paroxetine vs. age-matched Control*.

## Discussion

Use of antidepressants for the treatment of depressive disorders in adolescents is widespread, yet the impact of these drugs on the developing brain is still unclear. The present study investigated proteomic changes in the hippocampus of adolescent and adult rats treated chronically with the SSRI PRX. Differential changes in protein expression in adolescents compared to adults may provide clues as to why the adolescent brain responds differently to antidepressants, both in terms of reduced efficacy and increased risk of psychiatric side effects such as suicidal ideation. We have previously reported that adolescent rats treated with PRX as described in the current study fail to show an antidepressant-like behavioral pattern on the FST. In addition, adolescent rats treated with PRX show a significant reduction in social behavior and appear more susceptible than adults to increased anxiety in the early stages of SSRI treatment (Karanges et al., 2011). This elevation in adverse effects in adolescents is present despite substantially lower, although still therapeutically relevant (DeVane, 1999) plasma PRX in adolescents compared to adults.

**Figure 2:**
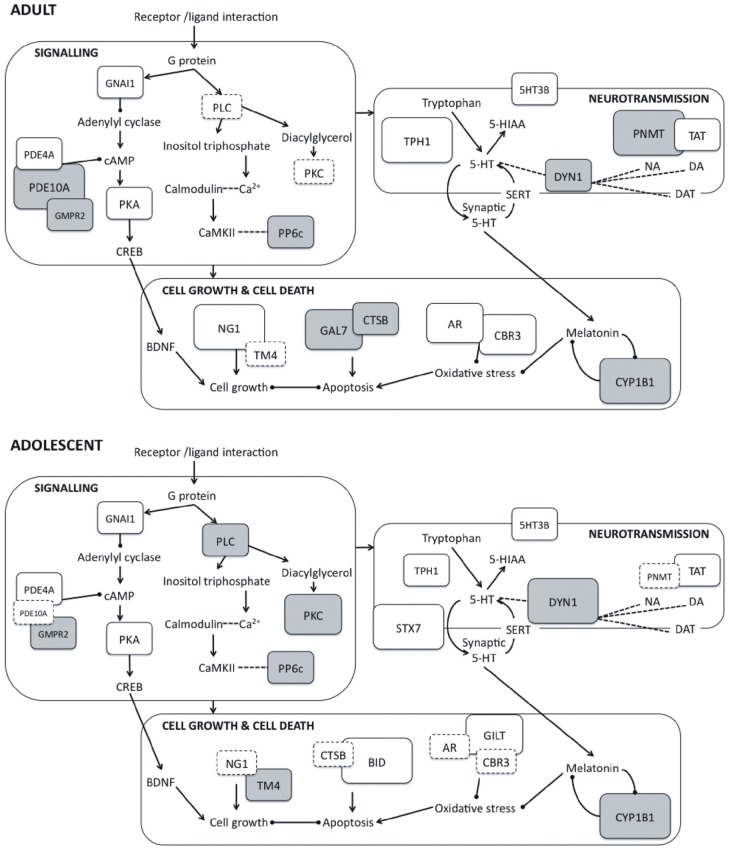
**Schematic representation of protein expression changes induced by paroxetine in adult (top panel) and adolescent rats (bottom panel) showing interactions between proteins and their involvement in key cellular activities.** Interaction between G protein-coupled receptors and their corresponding ligands leads to activation of downstream signaling pathways. Within these pathways, protein kinases such as PKA, PKC and CaMKII phosphorylate and regulate proteins impacting on cell growth and death, neurotransmission, syntaptic plasticity and receptor function. Arrows represent positive feedback or activation between protein units, while capped lines represent negative feedback or inhibition. The magnitude of the protein expression change induced by is represented by the size and color of the boxes: larger boxes represent larger fold changes, and downregulation is indicated with gray shading. Boxes with dashed outlines represent non-significant protein changes. 5-HIAA, 5-hydroxyindoleacetic acid; 5-HT, 5-hydroxytryptamine; 5HT3B, Serotonin 3B receptor; AR, Aldose reductase; BDNF, Brain-derived neurotrophic factor; BID, BH3-interacting domain death agonist; CaMKII, Calcium/calmodulin-dependent protein kinase; CBR3, Carbonyl reductase 3; CREB, cAMP response element binding protein; CTSB, Cathepsin B; CYP1B1: Cytochrome P450 1B1; DA, Dopamine; DAT, Dopamine transporter; DYN1, Dynamin 1; GAL7, Galectin 7; GILT, Interferon γ inducible protein 30; GMPR2, Guanosine monophosphate reductase 2; GNAI1, Guanine nucleotide-binding protein Gi, α1 subunit; NA, Noradrenaline; NG1, Neurogenin 1; PDE10A, Phosphodiesterase 10A; PDE4A, Phosphodiesterase 4A; PKA, Protein kinase A; PKC, Protein kinase C; PLC, Phospholipase C; PNMT, Phenylethanolamine-N-methyltransferase; PP6c, Protein phosphatase 6, catalytic subunit; SERT, Serotonin transporter; STX7, Syntaxin 7; TAT, Tyrosine aminotransferase; TM4, Tropomyosin 4; TPH1, Tryptophan hydroxylase 1.

Although several previous studies have investigated proteomic changes in adult rodents or neural cell cultures following the administration of PRX and other SSRIs (e.g., McHugh et al., 2008, 2010; Sillaber et al., 2008), this is the first study to our knowledge that has examined changes occurring in the adolescent brain. We provide evidence that chronic PRX treatment produces differential, although overlapping, changes in the hippocampal proteome in adolescent rats compared to adults, and these changes can be linked to the regulation of neurotransmitter function, major signaling pathways, cell proliferation and death, oxidative stress, and cytoskeleton structure. Figure 2 is a schematic proposing an overview of the major protein changes, their potential interactions and functional significance.

### Changes in proteins involved in regulation of neurotransmission

Tryptophan 5-hydroxylase 1 (TPH1) is the rate-limiting enzyme in 5-HT biosynthesis and alterations in this enzyme have been implicated in depression (Gizatullin et al., 2006), suicide attempts (Galfalvy et al., 2009), deliberate self-harm (Pooley et al., 2003) and the antidepressant-treatment response (Ham et al., 2007). The upregulation of TPH1 (>4-fold) in adults may in part underlie the positive treatment response in PRX-treated adults and the more equivocal therapeutic response in adolescents. Indeed, reduced expression of TPH1 has been associated with poorer response to PRX in other animal studies (Nakamura et al., 2006).

Only adult rats given PRX showed a significant decrease in phenylethanolamine N-methyltransferase (PNMT), an important enzyme in catecholamine biosynthesis and in the conversion of noradrenaline to adrenaline. PNMT transcription and activity is primarily regulated by glucocorticoids, with transcription increased in response to both chronic and acute stress (Sabban et al., 1995; Spasojevic et al., 2010). In addition, hypophysectomised animals, showing reduced corticosterone concentrations, show reductions in PNMT mRNA and activity (Jiang et al., 1989), suggesting that the reduction in PNMT expression shown here in adults may stem from the effects of PRX on the HPA axis. Indeed, reductions in HPA axis activity is a common response to antidepressant treatment, probably mediated by increases in glucocorticoid receptor (GR) expression and enhanced GR-mediated feedback inhibition (Pariante and Lightman, 2008), which may reduce PNMT expression. PNMT was unchanged here in adolescent rats treated with PRX, supporting the questionable efficacy of this drug on the developing brain.

Syntaxin 7 was upregulated almost 6-fold in PRX-treated adolescents but was below the detectable limit in adults. Syntaxin 7 is a transmembrane protein that forms a component of an endosomal SNARE complex, playing a role in the transport of vesicles containing neurotransmitters (Antonin et al., 2002; Rizo et al., 2006). PRX binds to and blocks SERT, preventing 5-HT reuptake, and thus potentially changes the rate of vesicular packaging and release of 5-HT.

### Changes in proteins linked to major signalling pathways

Expression of the signaling protein, protein kinase A (PKA), was increased (~2-fold) in both adolescents and adults treated with PRX. PKA is a serine/threonine kinase that plays an important role in the adenylate cyclase-cyclic adenosine monophosphate (cAMP) signaling system (Gould and Manji, 2002). PKA is activated in response to extracellular signals such as the binding of neurotransmitters to their receptors (Paez-Pereda, 2005). Once activated, PKA is involved in the phosphorylation and regulation of various cellular components including transcription factors, cytoskeletal proteins, ion channels and downstream signaling proteins and thereby plays a role in the regulation of cell survival, neurotransmitter production and release, receptor function and synaptic plasticity (Gould and Manji, 2002; Dwivedi and Pandey, 2011).

PKA may play an important role in depression and the therapeutic effects of antidepressants. Decreased PKA activity has been associated with suicide in both adult and adolescent populations (Dwivedi et al., 2004b; Pandey et al., 2005) and is observed in animal models of sub-chronic and chronic stress (Dwivedi et al., 2004a; Dwivedi and Pandey, 2011). In addition, long-term administration of PRX and other antidepressants modifies the distribution, cAMP-binding capabilities and activity of PKA (Popoli et al., 2001). Therapeutic effects arising from changes in PKA may be mediated by the downstream phosphorylation and activation of CREB, and subsequent transcription of BDNF, both of which have been associated with depression and the antidepressant response (Chen et al., 2001; Khundakar and Zetterström, 2006; Dwivedi and Pandey, 2011).

Upregulation of the inhibitory guanine nucleotide-binding protein (Gnai; Gi) in both age groups indicates a further modification of signaling pathways by PRX. Neurotransmitters and their receptors are linked to downstream signaling pathways such as the adenylate cyclase system via multi-subunit G proteins (Avissar and Schreiber, 2006). The various G protein subunits have different effects on downstream signaling, with adenylate cyclase activity stimulated by Gs and inhibited by Gi (Gould and Manji, 2002). Antidepressants are thought to produce changes in the activity and/or expression of both Gs and Gi subunits (Avissar and Schreiber, 2006), and have been reported to normalize reduced Gi levels in patients with depression (Avissar and Schreiber, 1994).

The purine nucleotides cAMP and cGMP are vital components in neuronal signaling pathways and tight regulation of these second messengers is required for maintenance of signaling integrity. Guanosine monophosphate reductase 2 (GMPR2) is involved in the recycling of adenosine and guanine nucleotides (Zhang et al., 2003) and phosphodiesterases (PDEs) are responsible for their degradation (Reierson et al., 2009). We demonstrate here PRX-induced modification in protein levels of GMPR2 and two PDEs, PDE10A (cAMP and cAMP-inhibited cGMP 3′,5′-cyclic phosphodiesterase 10A) and PDE4A (cAMP-specific 3′,5′-cyclic phosphodiesterase 4A). PDE4A, which was upregulated in both adults and adolescents treated with PRX, is specific for the degradation of cAMP (Reierson et al., 2009). Increased expression of PDE4A has been previously reported with a variety of antidepressant treatments (Takahashi et al., 1999; Esposito et al., 2009), and may be a homeostatic response to general upregulation of the cAMP signaling pathway (Takahashi et al., 1999). However, the antidepressant-like actions of the PDE4 inhibitor rolipram (Li et al., 2009) make it unlikely that PDE4A upregulation is directly responsible for antidepressant efficacy.

Downregulation (~6.5-fold) of PDE10A occurred in adult rats only. Unlike PDE4, PDE10 subtypes are important in the regulation of both cAMP and cGMP (Reierson et al., 2009) and polymorphisms in the gene encoding PDE10A have been associated with MDD in certain populations (Wong et al., 2006). Chronic administration of the PDE10A inhibitor papaverine has been associated with increases in hippocampal CREB, BDNF and trkB mRNA (Nibuya et al., 1996), suggesting a likely role of PDE10A in the antidepressant treatment response that is consistent with the effects of PRX noted here in adults.

Protein kinase C (PKC), a serine/threonine kinase, is a key component of the phosphoinositide signaling pathway (Gould and Manji, 2002). PKC is activated following stimulation of various receptors known to be affected by PRX and other SSRIs, including 5-HT_1A_, 5-HT_2C_ and the muscarinic M_1_ receptor (Hensler, 2003; Yamauchi et al., 2004; Carrasco and Sandner, 2005). Following activation, PKC phosphorylates downstream proteins involved in cell proliferation, cell differentiation, apoptosis, neurotransmitter release and downregulation of membrane receptor proteins (Nishizuka, 1992; Gould and Manji, 2002). We report here a decrease in PKC in adolescent rats (>3-fold), with no change apparent in adults. Altered PKC has been linked to suicide, particularly in adolescent populations (Pandey et al., 1997, 2004). PKC is also dysregulated in bipolar patients: Increased PKC signaling is observed during acute manic phases and decreased by chronic treatment with mood stabilizers (Zarate and Manji, 2009). Interestingly, phospholipase C, also decreased here in adolescents only, is a moderator of PKC activity (Nishizuka, 1992). Decreases in PKC and the related phosphoinositide signaling pathway may therefore be linked to the paradoxical response of some adolescents to antidepressants.

### Changes in proteins linked to neurogenesis and apoptosis

The aforementioned signaling pathways have downstream effects on cellular processes that influence cell growth and death. Differential changes in proteins implicated in neurotrophic and apoptotic pathways in adult and adolescent animals provide many clues as to the mechanisms underlying the response to PRX in adolescent humans.

PRX-treated adult rats showed reduced expression of pro-apoptotic proteins galectin-7 and cathepsin B, and dramatically increased expression (>4-fold) of the neurotrophic protein neurogenin-1, suggesting a general trophic and anti-apoptotic effect of PRX in adult animals. Galectin-7 is involved in the induction of p53-mediated apoptosis (Saussez and Kiss, 2006), while cathepsin B is a lysosomal cysteine protease implicated in a variety of apoptotic pathways (Guicciardi et al., 2000; Stoka et al., 2001). Various other studies have demonstrated anti-apoptotic and neurotrophic effects of antidepressant treatment including upregulation of survival (Murray and Hutson, 2007; Korsten et al., 2008) and neurotrophic proteins (Chen et al., 2001), and downregulation of pro-apoptotic proteins (Korsten et al., 2008). Indeed, the neurotrophic actions of antidepressants may be crucial for their therapeutic effects (Feldmann et al., 2007): Stimulation of neurogenesis is a key feature of many antidepressant therapies (Drzyzga et al., 2009; Malkesman et al., 2009; McKernan et al., 2009) and suppression of these neurotrophic actions can prevent the relief of depression-related symptoms (Santarelli et al., 2003).

In contrast to the neurotrophic and anti-apoptotic effects of PRX in adults, there were no appreciable positive changes in any of the aforementioned neurotrophic proteins in adolescents. This agrees with studies showing that fluoxetine has no effect on hippocampal neurogenesis in juvenile and peri-adolescent rats, despite stimulating neurogenesis in male adults (Hodes et al., 2009; Oh et al., 2009). In contrast, we observed dramatic increases (>4-fold) in expression of the pro-apoptotic protein BH3-interacting domain death agonist (BID) in treated adolescents. BID, a member of the Bcl-2 family of apoptotic proteins, acts by sequestering anti-apoptotic proteins such as Bcl-2 and activating pro-apoptotic family members, Bax and Bak (McKernan et al., 2009). Interestingly, the pro-apoptotic protein cathepsin B (decreased in adults) stimulates cleavage of BID, releasing cytochrome c from the mitochondria and triggering apoptosis (Stoka et al., 2001). Increases in apoptotic activity via BID may underlie the increased depression-like behaviors observed in some human adolescents treated with PRX, especially considering the recent demonstration of antidepressant effects of the BID inhibitor, BI-11A7 (Malkesman et al., 2009). Sufferers of MDD display brain features indicative of elevated apoptosis and decreased neurogenesis, such as reduced hippocampal volumes and downregulation of pro-survival proteins and neurotrophins (Drzyzga et al., 2009; McKernan et al., 2009). Furthermore, chronic stress, a risk factor for depression, can produce similar changes, suggesting that these effects may be causal in producing depression (McKernan et al., 2009).

### Changes in antioxidant proteins and proteins linked to oxidative stress

Antioxidant enzymes play a vital role in the protection of cells from oxidative stress. Production of oxidizing species is a normal consequence of a cell's metabolic processes, yet at high levels these species can damage intracellular components such as DNA, proteins and lipids (Ellis, 2007), disrupting signaling pathways and rendering the cell susceptible to apoptotic or necrotic cell death (Halliwell, 2001). The central nervous system is particularly susceptible to oxidative stress due to its high metabolic rate and unsaturated lipid content, and lower concentration of antioxidant enzymes compared to other tissues (Maser, 2006).

PRX-treated adults, but not adolescents, showed an upregulation (~3-fold) of two antioxidant enzymes, carbonyl reductase 3 (CBR3) and aldose reductase (AR), suggesting a possible neuroprotective effect of PRX on the adult but not the developing brain. CBR3, a member of the short chain dehydrogenase/reductase (SDR) family, catalyses the reduction of reactive aldehydes generated through lipid peroxidation (Maser, 2006). Lipid peroxidation and production of reactive oxygen species is elevated in patients with depression and in animal models of chronic stress (Bilici et al., 2001; Fontella et al., 2005), and many antidepressants appear to normalize antioxidant activity and markers of oxidative stress (Bilici et al., 2001; Zafir et al., 2009). Upregulation of CBR and/or AR likely have similar protective effects. Indeed, overexpression of a related CBR enzyme known as Sniffer in *Drosophila melanogaster* protects neurons against the effects of hyperoxia-induced oxidative stress (Botella et al., 2004). Similarly, overexpression of AR and related enzymes decreases the susceptibility of cells to apoptosis induced by oxidative stress by stimulating the reduction of reactive aldehydes (Ellis, 2007).

Three other proteins modified by PRX treatment are also linked to the production and/or reduction of cellular oxidative stress. Interferon gamma inducible protein 30 (GILT, also known as IFI30 or IP30) was upregulated almost 4-fold in adolescents, but absent in adults. GILT, a lysosomal thiol reductase, is associated with immune dysregulation and inflammatory disease (Satoh et al., 2008). Depression has also been associated with elevated immune function and secretion of inflammatory cytokines (Maes et al., 1994), thus GILT induction may be associated with the depressogenic effects of antidepressants in some adolescents. This increase in immune activation in MDD may be linked to oxidative stress: Immune activation stimulates production of reactive oxygen species (Bilici et al., 2001). GILT is also known to positively regulate expression, activity and stability of the antioxidant enzyme mitochondrial manganese superoxide dimutase (SOD2) (Bogunovic et al., 2008), hence GILT upregulation may be a compensatory response to the loss of antioxidant capabilities described above.

Cytochrome P450 1B1 (CYP1B1) was downregulated approximately 4-fold in both adolescents and adults treated with PRX. CYPs are phase I metabolising enzymes involved in the deactivation of endogenous compounds, drugs and environmental toxins (Miksys and Tyndale, 2002). Specifically, CYP1B1 is implicated in metabolism of the sterols 17β-estradiol and melatonin (Dutheil et al., 2008), both of which have neuroprotective roles, scavenging free radicals and protecting against oxidative stress (Goodman et al., 1996; Maharaj et al., 2006). Given that CYP1B1 is negatively regulated by melatonin (Chang et al., 2010), and both PRX and fluvoxamine increase melatonin levels in the brain (Härtter et al., 2001), it is conceivable that the downregulation of CYP1B1 might be mediated by effects of PRX on melatonin. Increases in synaptic 5-HT also stimulate melatonin production, inhibiting CYP1B1 activity (Nathan et al., 1998).

### Changes in cytoskeletal proteins

Dynamin-1 is a neuron-specific GTP-hydrolysing protein involved in synaptic vesicle endocytosis, neurotransmitter release and dopamine transporter (DAT) internalization (Mortensen et al., 2008; Wu et al., 2010). Many antidepressants, including PRX, are cationic amphiphillic drugs (CADs) that suppress the GTPase activities of dynamin (Rainey et al., 2010), inhibiting endocytosis and neurotransmitter release (Otomo et al., 2008). We report a marked decrease (6.89-fold) in dynamin-1 in PRX-treated adolescents, and a significant, but lesser, decrease in adults, suggesting that the effects of PRX on dynamin-1 may be exacerbated in adolescents. Interestingly, a study with sertraline associates the CAD-like qualities of the drug with its propensity to produce cellular toxicity in yeast cells (Rainey et al., 2010). The marked effect of PRX on dynamin-1 in adolescent rats may in part explain the increased expression of apoptotic proteins. Interestingly, CADs are also known to inhibit other phospholipid membrane-associated proteins such as phospholipases (Rainey et al., 2010).

### Limitations

2DE proteomic analysis is a powerful technique with the capacity to reveal potential targets for future research on the adolescent response to antidepressants. However, certain limitations of this technique must be considered when interpreting the current findings. Proteomics is an exploratory method that may result in false positives and often benefits from additional supportive analyses such as western blotting and/or PCR, which have not been conducted in the present study. As such, these findings should be interpreted with caution, particularly where fold changes do not exceed 1.5.

Furthermore, a shortcoming of the current study is the lower plasma PRX levels in adolescent rats compared to adults, despite provision of equivalent doses (Karanges et al., 2011). This caveat should be considered particularly where null results in adolescents accompany significant protein changes in adults. However, the presence of adverse behavioral effects (Karanges et al., 2011) and the heightened protein expression changes in adolescents (for example, in syntaxin 7, dynamin 1 and BID expression), suggest valid results despite lower plasma PRX.

## Conclusions

The present study demonstrates that chronic administration of PRX to adult and adolescent rats produces age-specific changes in the hippocampal proteome. Although similar changes were observed in many proteins in both age-groups, there were notable differences in the expression profiles of proteins implicated in apoptosis, oxidative stress, cytoskeletal structure, intracellular signaling and serotonergic and catecholaminergic neurotransmission. These findings, while suggestive rather than conclusive, demonstrate that the developing brain responds to PRX in a manner distinct from the adult brain, and provides some clues as to the mechanisms underlying the adolescent response to antidepressant drugs.

### Conflict of interest statement

The authors declare that the research was conducted in the absence of any commercial or financial relationships that could be construed as a potential conflict of interest.
